# The guiding value of the cinematic volume rendering technique in the preoperative diagnosis of brachial plexus schwannoma

**DOI:** 10.3389/fonc.2023.1278386

**Published:** 2023-12-13

**Authors:** Rui Chen, Yuncai Ran, Haowen Xu, Junxia Niu, Mengzhu Wang, Yanglei Wu, Yong Zhang, Jingliang Cheng

**Affiliations:** ^1^ Department of Magnetic Resonance, The First Affiliated Hospital of Zhengzhou University, Zhengzhou, China; ^2^ Department of Interventional Neuroradiology, The First Affiliated Hospital of Zhengzhou University, Zhengzhou, China; ^3^ MR Collaborations, Siemens Healthineers Ltd., Beijing, China

**Keywords:** brachial plexus schwannoma, diagnosis, magnetic resonance imaging, cinematic volume rendering technique, maximum intensity projection, surgical resection, preoperative diagnosis, eccentric embedding

## Abstract

This study aimed to explore and compare the guiding value of Maximum Intensity Projection (MIP) and Cinematic Volume Rendering Technique (cVRT) in the preoperative diagnosis of brachial plexus schwannomas. We retrospectively analyzed the clinical and imaging data of 45 patients diagnosed with brachial plexus schwannomas at the First Affiliated Hospital of Zhengzhou University between January 2020 and December 2022. The enhanced three-dimensional short recovery time inversion-recovery fast spin-echo imaging (3D-STIR-SPACE) sequence served as source data for the reconstruction of MIP and cVRT. Two independent observers scored the image quality and evaluated the location of the tumor and the relationship between the tumor and the brachial plexus. The image quality scores of the two reconstruction methods were compared using the nonparametric Wilcoxon signed-rank test, and the consistency between the image and surgical results was assessed using the weighted kappa. Compared to MIP images, cVRT images had a better performance of overall image quality (p < 0.001), nerve and lump visualization (p < 0.001), spatial positional relationship conspicuity (p < 0.001), and diagnostic confidence (p < 0.001). Additionally, the consistency between the cVRT image results and surgical results (kappa =0.913, P<0.001) was higher than that of the MIP images (kappa =0.829, P<0.001). cVRT provides a high guiding value in the preoperative diagnosis of brachial plexus schwannomas and is an important basis for formulating surgical plans.

## 1 Introduction

Brachial plexus schwannomas are benign tumors originating from the sheath membrane of the brachial plexus (1, 2). Brachial plexus schwannomas are mostly single and grow eccentrically, expand, and surround nerve fibers. The tumor usually pushes nerve bundles away. However, in some cases, nerve bundles may penetrate the tumor envelope with the tumor tightly attached to or wrapped around the nerve fibers. Surgical resection is the most effective treatment for brachial plexus schwannomas. Brachial plexus schwannoma surgery requires the surgeon to have a complete understanding of the anatomical structure and common anatomical variants encountered during the operation. In addition, the surgeon also needs to understand the clinical manifestations, pathological changes of the tumor, and related surgical techniques (3, 4). The local anatomical structures of the brachial plexus are complex, making the surgery technically challenging, and improper intraoperative management may result in brachial plexus injury and severe complications (5).The size, growth site, and biological behavior of brachial plexus schwannomas are closely associated with the formulation of clinical treatment methods. Therefore, accurate preoperative localization and qualitative diagnosis of tumors are crucial for clinicians.

Magnetic resonance imaging (MRI) is the best non-invasive examination method for diagnosing the brachial plexus because of its high signal-to-noise ratio and high tissue contrast (6). In recent years, brachial plexus imaging has received increasing attention due to the widespread use of magnetic resonance imaging (7, 8). Brachial plexus imaging can accurately describe the imaging characteristics of brachial plexus schwannomas, including the lesion size, location, source, and surrounding tissue involvement, to guide surgical methods and evaluate resectability (9, 10). The 3D-STIR-SPACE sequence can display the structures of the brachial plexus by inhibiting background fat, which helps to diagnose the location, origin, and extent of the brachial plexus schwannoma (11–13). Additionally, it can be reconstructed in three dimensions through post-processing to clearly show the spatial relationship between the tumor and the brachial plexus. Currently, this is the preferred method for evaluating the brachial plexus. MIP is the most commonly used two-dimensional slice direction reconstruction method (14, 15). MIP images have the advantages of an intuitive, comprehensive, and overall display of the relationship between the brachial plexus schwannoma and brachial plexus in the imaging range. As a new image 3D visualization technology, cVRT can simulate the interaction and propagation characteristics of light rays while passing through 3D data (16). Compared to traditional MIP, it can obtain more realistic 3D images (17).

This study preliminarily attempted to apply cVRT to the preoperative diagnosis of brachial plexus schwannomas, using surgical results as a reference, to explore the guiding value of cVRT compared to traditional MIP reconstruction methods in the diagnosis and evaluation of brachial plexus schwannomas and the spatial relationship between lesions and surrounding structures, and to provide more intuitive and accurate imaging information for clinical practice.

## 2 Materials and methods

### 2.1 Study participants

**Figure 1:**
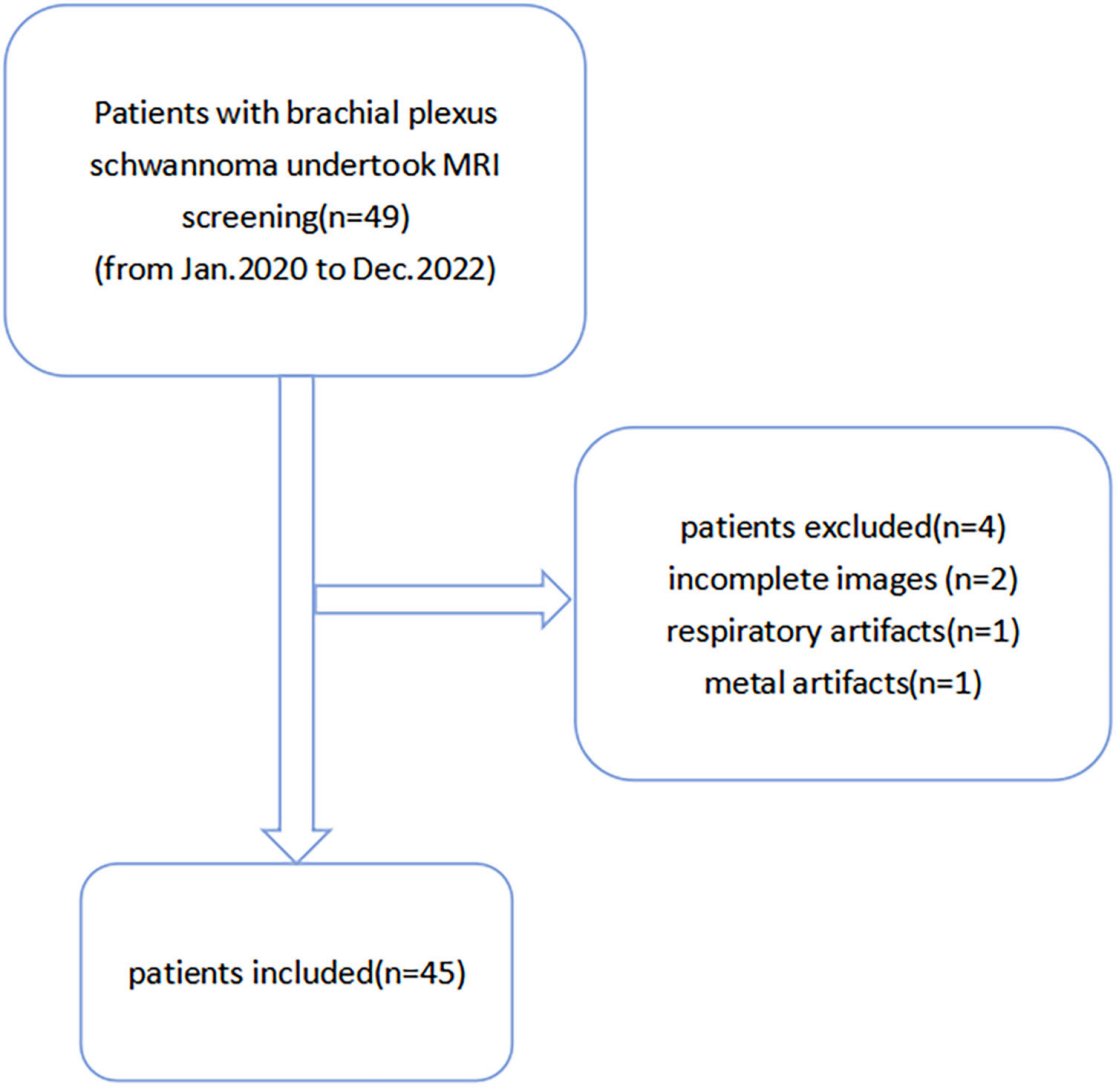
Participant selection flowchart.

Forty-nine patients with brachial plexus schwannomas at the First Affiliated Hospital of Zhengzhou University between January 2020 and December 2022 were retrospectively collected. Four patients were excluded because of incomplete magnetic resonance imaging, respiratory artifacts, or metal artifacts. A total of 45 patients were included in this study. A flowchart of patient enrollment is shown in **Figure 1**. All patients underwent MR examinations, including T1-weighted imaging (T1WI), T2WI, 3D-STIR-SPACE, and enhanced T1WI within one week before surgery. This study protocol was reviewed and approved by the Ethics Committee of First Affiliated Hospital of Zhengzhou University, approval number 2019-KY-231,and all patients provided informed permission.

### 2.2 MRI parameters

All patients were examined using a 3T MR scanner (MAGNETOM Lumina, Siemens Healthineers, Erlangen, Germany). A 64-channel head and neck coil and a 16-channel body coil are used, both of which are placed partially overlapping. The participants were placed in the supine position with the head and neck raised appropriately and their arms placed on both sides. The patients avoided deep breathing and swallowing throughout the process to minimize motion artifacts. Conventional scanning sequences included coronal turbo spin echo (TSE) T1WI; transverse, coronal, and sagittal TSE T2WI; coronal 3D SPACE-STIR T2WI; and T1WI-enhanced sequences. The contrast agent used was domestic gadolinium pentanoate meglumine (Gadolinium-DTPA) at a dose of 0.2 mmol/kg and was rapidly injected intravenously through a high-pressure syringe. Coronary 3D SPACE-STIR T2WI starts scanning three minutes after injecting the contrast agent. Coronal scanning covers the anterior and posterior edges of the spinal canal, the upper edge of the second cervical vertebral body, the upper edge of the second thoracic vertebral body, and the bilateral humeral heads. Axial scanning refers to the coronal position, covering the fifth cervical nerve and the first thoracic nerve root distribution on both sides. The detailed acquisition parameters for the MRI sequences are listed in **Table 1**.

**Table 1:** Scanning sequence and main parameters of the brachial plexus.

Scanning sequence	FOV (mm^2^)	matrix	Slice thickness (mm)	TR/TE (ms)
T1WI	400×400	307 × 307	4.0	650/12
T2WI	220×220	314 × 314	4.0	3000/101
3D-STIR-SPACE	420×420	466 × 466	3.0	3000/160
T1WI+C	400×400	307 × 307	4.0	650/12

FOV, Field of view; TR, Time of repetition; TE, Time of echo.

### 2.3 Image analysis

The 3D-STIR-SPACE images were transmitted to a post-processing workstation (Syngo.via 4.0; Siemens Healthineers, Erlangen, Germany) for MIP and cVRT reconstruction. After MIP reconstruction of the brachial plexus, soft tissues, such as muscles, were subtracted according to clinical needs to reduce interference with the anatomical positional relationship between the tumor and the brachial plexus. Multiplanar reconstruction (MPR) was used to delineate the tumor range slice-by-slice. cVRT technology was used to render the brachial plexus and tumor in different colors and obtain a three-dimensional model. The positional relationship and tissue structure of the brachial plexus and tumor are displayed in all directions.

### 2.4 Observation indicators

Two radiologists, Yong Zhang, and Yuncai Ran, with 13 and 8 years of experience in MR neurography, respectively, randomly analyzed the images. They were blinded to the patient’s clinical information, sequence parameters, and final radiological reports of the MR neurography. Radiologists assessed the location, size, shape, and magnetic resonance signal characteristics of brachial plexus schwannomas, as well as the spatial relationship between the tumors and brachial plexus nerves from different angles in all patients. Additionally, they used a 4-point system, as described in **Table 2**, to evaluate the image quality. The highest qualitative score was defined as the ability to clearly distinguish the spatial positional relationship between the brachial plexus sheath tumor and the brachial plexus nerve. One radiologist, Yuncai Ran, repeated the measurements 2 weeks later to assess the intra-observer agreement.

**Table 2:** The four-point qualitative scoring system, utilized for assessing the MIP and cVRT images.

categories	1	2	3		4
Overall image quality	Poor quality	Moderate quality	Good quality		perfect quality
Background suppression	Poor suppression	Moderate suppression	Good suppression		Perfect suppression
Nerve and lump Visualization	No visualization	Many partial visualizations	Few partial visualizations		Full visualization
Spatial positional relationship Conspicuity	No conspicuity	Poor conspicuity	Moderate conspicuity		Excellent conspicuity
Diagnostic Confidence	No confidence	Low confidence	Intermediate confidence		High confidence

MIP, Maximum Intensity Projection; cVRT, Cinematic Volume Rendering Technique.

### 2.5 Surgical records

Surgery was performed under general anesthesia. An incision was made to explore the brachial plexus centered on the tumor. After the tumor was exposed, nerve bundles that flattened and dispersed around the tumor were carefully observed. The area with the lowest number of nerve bundles was selected. Along the direction of the nerve bundle walking, the fibrous tissue that wrapped the tumor was cut layer-by-layer until the real tumor capsule. The nerve bundle and schwannoma were separated, and the tumor was completely removed. All 45 patients were operated on by the same experienced neurosurgeon who judged the relationship between the brachial plexus schwannoma and the brachial plexus during the operation. All tumor tissues were sent for pathological examination after surgery, and all were confirmed as benign schwannomas.

### 2.6 Statistical analysis

All analyses were performed using the statistical software package (SPSS Inc. (Chicago, IL, USA). The image quality scores of the MIP and cVRT images were compared and statistically analyzed using the nonparametric Wilcoxon signed-rank test. Inter- and intraobserver agreements were assessed using intraclass correlation coefficients (ICC). Considering the judgment results of surgery as the gold standard, the two imaging modalities were compared with the surgical results. ICC was used to determine the consistency of tumor size, and weighted kappa was used to determine the consistency of tumor location and spatial location between the tumors and brachial plexus nerves. The ICC model was based on a two-way random comparison of absolute agreement types, and the coefficients were computed at a significance level of 5%. The K values between 0 to 0.2, 0.2 to 0.4, 0.4 to 0.6, 0.6 to 0.8, and 0.8–1.0 indicated poor, fair, moderate, substantial, and almost perfect agreements, respectively. P < 0.05 was considered statistically significant.

## 3 Results

### 3.1 General data

The 45 patients included 24 male and 21 female participants, aged 18-79 years, with an average of 37.5 years. The shortest course was 15 days, and the longest was 18 years, with an average of 4.5 years. Among them, 26 had a course of> 1 year. Among the 45 patients, 35 had neck and axillary masses as the main clinical symptoms, five had limb numbness or weakness, three had neck or upper limb pain, and two had no obvious symptoms (**Table 3**). Among the 45 cases, tumors were situated in the supraclavicular portion in 35 cases, and in the infraclavicular portion in 10 cases. The tumor originated from the C5 and 6 nerve roots of the brachial plexus in 7 cases, the C8 nerve root in 3 cases, the superior trunk in 6 cases, the middle trunk in 12 cases, the interior trunk in 7 cases, the lateral cord in 3 cases, posterior cord in 2 cases, medial cord in 4 cases, and terminal branches in 1case. The minimum tumor body was 1.5 cm × 1.0 cm × 0.8 cm, and the maximum tumor body was 10.0 cm × 7.8 cm × 4.0 cm.

**Table 3:** Demographic characteristics of study population.

Characteristic	All Participants (n=45)
Age, years	37.5 (18–79)
Sex	
Female	24(53.33%)
Male	21(46.66%)
Main clinical symptoms	
Lumps in the neck or underarm on the affected side	35(77.77%)
Numbness in the hands	5(11.11%)
Swelling and weakness of the neck and upper limbs	3(6.66%)
No obvious symptoms	2(4.44%)

### 3.2 Imaging characteristics

**Figure 2:**
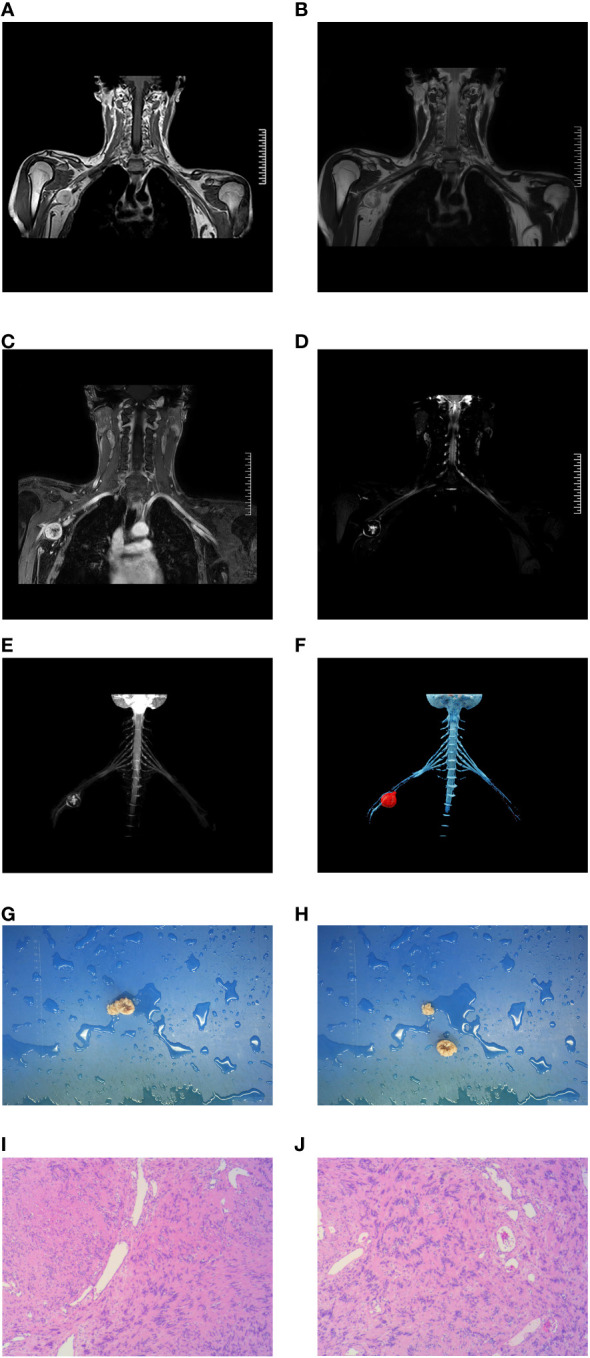
A 17-year-old man presenting with a a circular mass at the beginning of the right axillary horizontal ulnar nerve can be seen in **(A-F)**. The pathological results in **(G, H)** show that the section is gray and yellow, and is considered a schwannoma **(I, J)**.

The maximum diameters of all the masses ranged from 1.5 to 10.0 cm, with an average of 4.1 cm. The tumors of 45 patients showed unilateral growth along the brachial plexus with a fusiform, spherical, or oval shape. In 45 cases of brachial plexus, schwannomas showed isosignal or slightly low signal intensity on T1-weighted images and heterogeneous high signal intensity on T2-weighted images. After enhancement, the signal was evenly or unevenly strengthened, and a low-signal area appeared in the middle of the large mass, such as **Figures 2A-C**.

### 3.3 Results of the four-point qualitative scoring system

**Figure 3:**
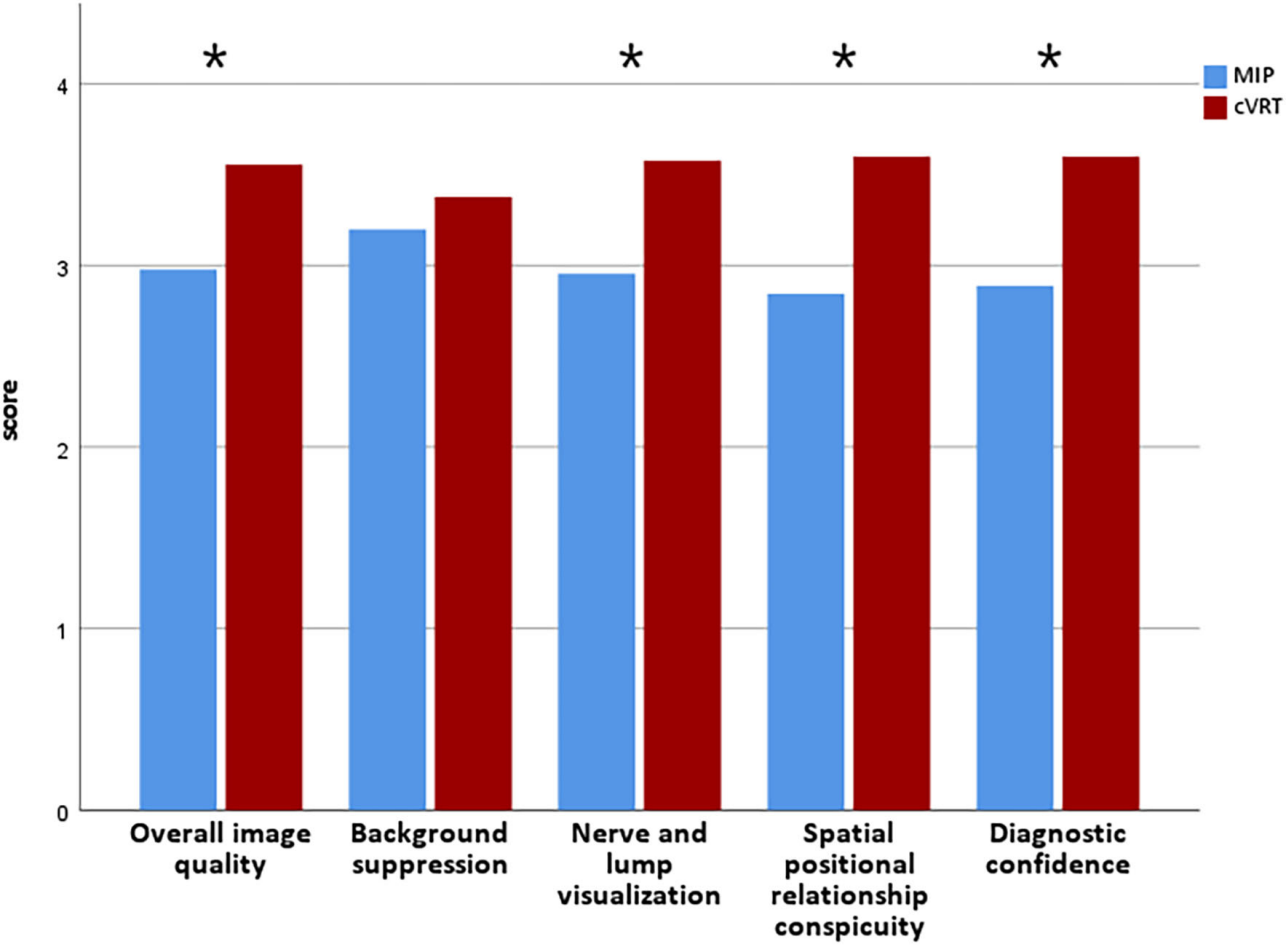
Quantitative score distribution of 2 MRI sets. Blue represents MIP, red represents cVRT, * denotes statistically significant differences (p< 0.05), and p-values refer to the results of the nonparametric Wilcoxon signed-rank test.

The image quality scores of the two radiologists revealed better overall image quality (p< 0.001), nerve and lump visualization (p< 0.001), spatial positional relationship conspicuity (p< 0.001), and diagnostic confidence (p< 0.001) when examining cVRT images than when examining MIP images. No significant differences were found in background suppression (**Figure 3**). The ICC for inter- and intra-rater agreements of the MIP and cVRT images are shown in **Table 4**.

**Table 4:** Intra-observer and inter-observer agreement assessment of interpretations of 2 MRI sets.

Metric	Intra-observer Agreement			Inter-observer Agreement		
	MIP		cVRT	MIP		cVRT
Overall image quality	0.905 (0.834-0.947)		0.921 (0.859-0.956)	0.886 (0.802-0.936)		0.915 (0.849-0.952)
Background suppression	0.860 (0.758-0.920)		0.898 (0.823-0.943)	0.851 (0.740-0.916)		0.892 (0.813-0.939)
Nerve and lump visualization	0.852 (0.746-0.915)		0.920 (0.859-0.955)	0.803 (0.669-0.887)		0.910 (0.842-0.950)
Spatial positional relationship conspicuity	0.765 (0.610-0.864)		0.967 (0.941-0.982)	0.739 (0.573-0.847)		0.935 (0.884-0.964)
Diagnostic confidence	0.888 (0.806-0.937)		0.933 (0.881-0.963)	0.868 (0.772-0.925)		0.932 (0.879-0.962)

MIP, Maximum Intensity Projection; cVRT, Cinematic Volume Rendering Technique.

### 3.4 Agreement analysis between imaging results and surgical results

The sizes and locations of the tumors revealed using MIP and CVRT were consistent with those observed intraoperatively. In this study, the spatial positional relationship between the brachial plexus schwannoma and the brachial plexus nerve was mainly characterized by eccentric embedding, pushing, and embedding and pushing. In the surgical results, 35 patients had eccentrically embedded brachial plexuses, 6 patients had tumors pushing against the brachial plexus, and 4 patients had tumors both embedded and pushing against the brachial plexus. The evaluation of cVRT-reconstructed images based on the 3D-STIR-SPACE sequence revealed that 37 patients had eccentrically embedded brachial plexuses, two patients had tumors pushing against the brachial plexus, and six patients had tumors both embedding and pushing against the brachial plexus, the example was shown in **Figure 2**. The agreement between MIP and surgical results was good (kappa = 0.829, P < 0.001), and cVRT had a higher agreement with the surgical results (Kappa = 0.913, P < 0.001) (**Table 5**).

**Table 5:** Results of interpretations of 2 MRI sets compared with surgery in all 45 patients.

Observation indicators	MIP	cVRT	Surgery	MIP *vs* Surgery	cVRT *vs* Surgery
Size (cm)	4.13 ± 1.62	3.98 ± 1.64	4.05 ± 1.70	0.985^a^	0.990^a^
Location				1.000^b^	1.000^b^
C5 and C6 nerve roots	7	7	7		
C8 nerve root	3	3	3		
Superior trunk	6	6	6		
Middle trunk	12	12	12		
Interior trunk	7	7	7		
Lateral cord	3	3	3		
Posterior cord	2	2	2		
Medial cord	4	5	5		
Terminal branches	1	1	1		
Spatial relationship				0.829^b^	0.913^b^
Eccentric embedding	37	36	35		
Pushing	2	4	6		
Embedding and pushing	6	5	4		

MIP, Maximum Intensity Projection; cVRT, Cinematic Volume Rendering Technique.

^a^represents intraclass correlation coefficients (ICC), and ^b^represents the weighted kappa test.

## 4 Discussion

The brachial plexus is located superficially, with complex anatomy, making it susceptible to various diseases such as trauma and tumors. Therefore, accurate localization and qualitative diagnosis of brachial plexus lesions are crucial for clinical treatment. For patients with brachial plexus schwannoma affecting brachial plexus function, the most effective clinical treatment approach is surgical resection (18).The surgery is difficult because of the complex local anatomy of the brachial plexus (19) and can easily cause high-level damage to the nerve, resulting in serious upper extremity dysfunction. Therefore, preoperative diagnosis of brachial plexus schwannoma through imaging examinations is of utmost importance.

MRI is the preferred imaging modality for evaluating the anatomical structure and pathology of the brachial plexus (6), but there is no consensus on the most appropriate protocol for brachial plexus MRI (20).The brachial plexus is composed of the anterior branches of the 5th to 8th cervical nerves and the anterior branch of the 1st thoracic nerve. Due to the non-collinear and non-coplanar nature of the brachial plexus nerve, in order to achieve complete visualization of the brachial plexus nerve, the scanning range in this study extends from the upper edge of the second cervical vertebra to the upper edge of the second thoracic vertebra. The anterior region includes the anterior edge of the vertebral body, the posterior region includes the posterior edge of the vertebral canal, and both sides include the humeral heads.

In 1993, Fler et al. (21) first reported magnetic resonance neurography (MRN), and its application has gradually gained clinical recognition. The commonly used methods include diffusion-weighted imaging with background body signal suppression (DWIBS) and 3D-STIR-SPACE. DWIBS is a background suppression diffusion sequence that combines fat suppression with diffusion-weighted imaging techniques. By adding background suppression to the diffusion imaging, it can reduce motion artifacts caused by respiration, suppress fat tissue signals, increase tissue image contrast, and improve scanning results. However, it is prone to susceptibility artifacts due to magnetic field inhomogeneity (22). The 3D-STIR-SPACE sequence can selectively suppress the signals of fat and muscles, both around and within the nerves, by using enhanced fat suppression and inversion recovery techniques. This creates a clear contrast between the nerves and surrounding tissues, allowing for a clear imaging of the brachial plexus. It is currently the most mature sequence for clinical brachial plexus examinations (23). Furthermore, some studies have shown that the DIXON method of fat suppression is considered advantageous as it aims to achieve uniform fat suppression with less susceptibility artifacts. It also increases the visibility of the nerves without prolonging the examination time (24). However, it has not been widely applied in clinical practice to date. In this study, the bilateral brachial plexus can be clearly, intuitively and three-dimensionally displayed by using 3D-STIR-SPACE sequence combined with enhanced scanning on a 3.0T high-field magnetic resonance scanner. This allows for accurate localization and diagnosis of tumors and other diseases affecting the brachial plexus, as well as assessment of the site and degree of nerve injury. It helps clinicians choose appropriate treatment plans and surgical techniques (25).

The use of cVRT in brachial plexus nerve imaging has rarely been reported. This study uses cVRT on the basis of enhanced 3D-STIR-SPACE sequence, which brings new inspiration to clinical work.cVRT has higher accuracy than MIP reconstruction technology in judging the positional relationship between the brachial plexus schwannoma and the brachial plexus nerve. It also has high application value in preoperative surgical plan formulation and the prediction of surgical complications.

It is difficult to evaluate brachial plexus injury solely using 3D-SPACE-STIR thin-section images, which are prone to misjudging the relationship between brachial plexus schwannoma and the brachial plexus because of the complexity of the anatomical structure of the brachial plexus. The MIP reconstruction encodes and projects the maximum intensity value of each pixel on each path in the volume scanning data. It is widely used in tissues and structures with relatively high density, such as blood vessels, bones, and strengthened soft tissue lesions. Although the 3D-STIR-SPACE sequence is isotropic and can be viewed in a 360° rotation in MIP, MIP represents a maximum intensity projection, and each angle presents a 2D image, which makes it difficult to discern finer details and accurately determine the spatial relationship between the brachial plexus and the tumor. However, cVRT uses multiple light sources to create a 3D effect, which creates a spatial positional relationship between the brachial plexus and brachial plexus schwannoma.

This study demonstrated that the overall image quality, nerve and lump visualization, spatial positional relationship conspicuity, and diagnostic confidence of cVRT were better than those of MIP. This may be because cVRT is a realistic cinematic rendering technology based on a precise physical simulation of the interaction between light and matter. It uses multiple light sources to create interactions between light and human tissues (reflection, refraction, primary scattering, secondary scattering, etc.), enriching and enhancing depth, forming perceptions, and forming more realistic shadows (26). It has the advantage of displaying soft tissue anatomical slices and blood vessels more clearly and accurately. Contrarily, the three-dimensional anatomical effect tends to be more realistic, providing more detailed and accurate anatomical information for clinical use (27–29). cVRT selects the brachial plexus schwannoma for MRP reconstruction and delineates it layer-by-layer based on the MIP technology. It then rendered the tumor and brachial plexus in different colors to improve the sharpness of the tissue edge without redundant background interference. In addition, some studies have also shown that the main innovative imaging reconstruction techniques, 3D modeling technologies (CAD, VR, AR), and 3D printing applications can be helpful in the future preoperative planning of surgery for pediatric tumors (30, 31).Therefore, cVRT can accurately show the positional relationship between the tumor and the brachial plexus, which is very important for evaluating its risk, preventing complications, and formulating treatment plans.

However, cVRT has certain limitations. First, similar to other 3D reconstruction algorithms, the final reconstructed image quality of cVRT depends on the original image quality. Therefore, a reduction in the original image quality, such as artifacts caused by physical, patient-related, and scanning machines, will reduce the image quality of the 3D reconstruction. Some studies have shown that diffusion tensor imaging (DTI) can be used to characterize peripheral nerves (32, 33). DTI has a higher resolution and has more advantages for the display of damaged nerve bundles.cVRT based on DTI may be more advantageous for showing the spatial positional relationship between brachial plexus schwannoma and brachial plexus, which needs to be verified in future studies. Second, cVRT, a more complex VR reconstruction technology, requires layer-by-layer delineation of the tumor range after tumor MPR reconstruction. Therefore, the reconstruction time is longer than the traditional 3D reconstruction time, and the high requirements for post-processing imageability limit the popularization of this technology to a certain extent. We can effectively reduce the reconstruction time by updating the cVRT post-processing software so that it can intelligently outline the tumor boundaries. Finally, owing to the novelty of this technology, large-scale multicenter clinical studies are needed to verify whether it can significantly improve diagnostic accuracy.

## 5 Conclusion

cVRT based on 3D-STIR-SPACE has great practical significance for preoperative diagnosis of clinical brachial plexus schwannomas. cVRT is superior to MIP, and it is recommended to be actively carried out in practice to improve the accuracy of the preoperative diagnosis of brachial plexus schwannoma and to better assist in the formulation of surgical plans.

## Data availability statement

The original contributions presented in the study are included in the article/**Supplementary Material**. Further inquiries can be directed to the corresponding author.

## Ethics statement

The studies involving humans were approved by Ethics Committee of the First Affiliated Hospital of Zhengzhou University. The studies were conducted in accordance with the local legislation and institutional requirements. The participants provided their written informed consent to participate in this study.

## Author contributions

RC: Conceptualization, Data curation, Formal analysis, Investigation, Methodology, Project administration, Resources, Software, Supervision, Validation, Visualization, Writing – original draft, Writing – review & editing. YR: Data curation, Methodology, Supervision, Writing – original draft, Writing – review & editing. HX: Data curation, Methodology, Supervision, Writing – review & editing. JN: Data curation, Methodology, Supervision, Writing – review & editing. MW: Methodology, Software, Supervision, Writing – review & editing. YW: Methodology, Software, Supervision, Writing – review & editing. YZ: Methodology, Supervision, Writing – review & editing. JC: Methodology, Project administration, Resources, Supervision, Writing – review & editing.
